# Technical Note: Consistency of PTW30013 and FC65‐G ion chamber magnetic field correction factors

**DOI:** 10.1002/mp.13623

**Published:** 2019-06-17

**Authors:** S. J. Woodings, B. van Asselen, T. L. van Soest, L. A. de Prez, J. J. W. Lagendijk, B. W. Raaymakers, J. W. H. Wolthaus

**Affiliations:** ^1^ Department of Radiotherapy University Medical Center Utrecht Heidelberglaan 100 Utrecht 3584CX The Netherlands; ^2^ VSL – Dutch Metrology Institute Delft The Netherlands

**Keywords:** dosimetry, FC65‐G, magnetic field, MRI‐Linac, PTW30013

## Abstract

**Purpose:**

Reference dosimetry in a strong magnetic field is made more complex due to (a) the change in dose deposition and (b) the change in sensitivity of the detector. Potentially it is also influenced by thin air layers, interfaces between media, relative orientations of field, chamber and radiation, and minor variations in ion chamber stem or electrode construction. The PTW30013 and IBA FC65‐G detectors are waterproof Farmer‐type ion chambers that are suitable for reference dosimetry. The magnetic field correction factors have previously been determined for these chamber types. The aim of this study was to assess the chamber‐to‐chamber variation and determine whether generic chamber type‐specific magnetic field correction factors can be applied for each of the PTW30013 and FC65‐G type ion chambers when they are oriented anti‐parallel (ǁ) to, or perpendicular (⊥) to, the magnetic field.

**Methods:**

The experiment was conducted with 12 PTW30013 and 13 FC65‐G chambers. The magnetic field correction factors were measured using a practical method. In this study each chamber was cross‐calibrated against the local standard chamber twice; with and without magnetic field. Measurements with 1.5 T magnetic field were performed with the 7 MV FFF beam of the MRI‐linac. Measurements without magnetic field (0 T) were performed with the 6 MV conventional beam of an Elekta Agility linac. A prototype MR‐compatible PTW MP1 phantom was used along with a prototype holder that facilitated measurements with the chamber aligned 90° counter‐clockwise (⊥) and 180° (ǁ) to the direction of the magnetic field. A monitor chamber was also mounted on the holder and all measurements were normalized so that the effect of variations in the output of each linac was minimized. Measurements with the local standard chamber were repeated during the experiment to quantify the experimental uncertainty. Recombination was measured in the 6 MV beam. Beam quality correction factors were applied. Differences in recombination and beam quality between beams are constant within each chamber type. By comparing the results for the two cross calibrations the magnetic field correction factors can be determined for each chamber, and the variation within the chamber‐type determined.

**Results:**

The magnetic field correction factors within both PTW30013 and FC65‐G chamber‐types were found to be very consistent, with observed standard deviations for the PTW30013 of 0.19% (ǁ) and 0.13% (⊥), and for the FC65‐G of 0.15% (ǁ) and 0.17% (⊥). These variations are comparable with the standard uncertainty (k = 1) of 0.24%.

**Conclusion:**

The consistency of the results for the PTW30013 and FC65‐G chambers implies that it is not necessary to derive a new factor for every new PTW30013 or FC65‐G chamber. Values for each chamber‐type (with careful attention to beam energy, magnetic field strength and beam‐field‐chamber orientations) can be applied from the literature.

## 1 Introduction

Elekta AB (Stockholm, Sweden), Philips (Best, The Netherlands) and University Medical Center Utrecht have developed a linear accelerator (linac) with integrated 1.5 T magnetic resonance imaging (MRI). This combination facilitates simultaneous irradiation and high‐precision image guidance with soft‐tissue contrast.^1^ The Elekta Unity MRI‐linac is an upgraded version of the machine described by Raaymakers et al.^2^ The magnetic field (*B*) points out of the entrance of the bore, and is at all times at 90^o^ to the central axis of the radiation beam delivered from the linac mounted on its ring gantry.

The magnetic field affects the dose deposition within a phantom and patient within the MRI‐linac. The Lorentz force acts on charged particles, pulling them in a direction orthogonal to their motion and the magnetic field, which perturbs the dose deposition kernel.^3^ The Lorentz force affects the trajectories of charged particles into, and within, a radiation detector. In addition, seemingly incidental environmental conditions such as thin air layers around the outside of an ion chamber or minor variations in ion chamber stem or electrode construction, may also make significant changes to ion chamber readings.^4^ Previously the performance of Farmer‐type chambers have been investigated and their feasibility confirmed.^5^, ^6^, ^7^, ^8^

Calibration of the output of a linac beam requires accurate measurement of absorbed dose to water. National and international codes of practice specify what equipment should be used and how these measurements should be performed.^9^, ^10^, ^11^, ^12^ Dosimetry on a clinical system must be traceable to an internationally‐accepted primary standard. Dosimetry factors for the local standard are usually transferred to field detectors through cross‐calibration. For the MRI‐linac additional factor(s) are required due to the magnetic field. Various definitions and values of the magnetic field correction factor have been published.^6^, ^13^, ^14^, ^15^, ^16^, ^17^ Measuring the magnetic field correction factor for an individual chamber is a nontrivial activity involving high‐precision measurements with and without magnetic field, and potentially with multiple relative orientations of radiation beam, magnetic field and ion chamber. If it can be established that there is little intra‐chamber‐type variation, then generic chamber‐type values from the literature, or in the future from a code of practice, can be reliably used in the clinic.

The aim of this study was to assess the chamber‐to‐chamber variation in the magnetic field correction factors and determine whether generic chamber type‐specific values can be applied for each of the PTW30013 and FC65‐G ‐type ion chambers when they are oriented anti‐parallel (ǁ) to, and perpendicular (⊥) to, the magnetic field.

## 2 Theory

Two cross‐calibrations can be performed, one with and one without magnetic field, so that the magnetic field correction factor can be determined.

The Dutch code of practice for reference dosimetry (NCS 18),^9^ consistent with AAPM 51^10^, ^12^ and IAEA TRS 398,^11^ describes the calculation of dose to water from a megavoltage photon beam of quality *Q* as:

(1) $$D_{w,Q}=M_{Q}\cdot N_{D,w,Q_{0}}\cdot k_{Q,Q_{0}}$$

where *M*_Q_ is the ion chamber reading corrected for influence quantities such as recombination, polarity, temperature and pressure, *N*_D,w,Q0_ is the absorbed dose to water calibration coefficient for the ion chamber at beam quality *Q*_0_ and *k*_Q,Q0_ is the beam quality correction factor.

Within a strong magnetic field, the charged particles follow curved trajectories in the medium, and into and within the chamber, and thus the relationship between charge collected within the ion chamber and dose deposited in water is also dependent upon *B* and the relative orientations of magnetic field, radiation beam and chamber.

This effect can be incorporated into the reference dosimetry in a number of ways,^6^, ^16^, ^17^ which are all consistent with:

(2) $$D_{w,Q,B}=M_{Q,B}\cdot N_{D,w,Q_{0}}\cdot k_{Q,Q_{0}}\cdot k_{B}$$

The cross calibration (in a magnetic field) of a field ion chamber to the local standard ion chamber is based on the equality of absorbed dose at the dosimetric reference point:

(3) $$N_{D,w,Q_{0}}^{\text{field}}\cdot k_{Q,Q_{0}}^{\text{field}}\cdot k_{B}^{\text{field}}=N_{D,w,Q_{0}}^{\text{ref}}\cdot k_{Q,Q_{0}}^{\text{ref}}\cdot k_{B}^{\text{ref}}\cdot \frac{M_{Q,B}^{\text{ref}}}{M_{Q,B}^{\text{field}}}$$

In general, generic *k*_Q,Q0_ factors would be used from the standard code of practice, although some centers may have chamber‐specific *k*_Q,Q0_ factors from primary standards laboratories that offer calibrations with beams other than Co‐60.

If a cross‐calibration is also performed with *B* = 0 T (on an Elekta Agility linac in this study), then the magnetic field correction factor can be determined from the combination of cross calibrations with beam quality *Q*_1_ and field *B* = 0 T, and beam quality *Q*_2_ and field *B* = 1.5 T, along with the known magnetic field correction factor of the local standard chamber.

(4) $$k_{B}^{\text{field}}=k_{B}^{\text{ref}}\cdot \frac{M_{Q_{2},B}^{\text{ref}}}{M_{Q_{2},B}^{\text{field}}}\cdot \frac{k_{Q_{2},Q_{0}}^{\text{ref}}}{k_{Q_{2},Q_{0}}^{\text{field}}}\cdot \frac{M_{Q_{1}}^{\text{field}}}{M_{Q_{1}}^{\text{ref}}}\cdot \frac{k_{Q_{1},Q_{0}}^{\text{field}}}{k_{Q_{1},Q_{0}}^{\text{ref}}}$$

It has been shown previously that generic *k*_Q,Q0_ factors are applicable for these chamber types.^18^ If generic *k*_Q,Q0_ factors are used and a comparison is made between chambers that are all the same type, then the ratios of *k*_Q,Q0_ are all constants and have no effect on the distribution of $k_{B}^{\text{field}}$ values. The $k_{B}^{\text{ref}}$ is also unchanging throughout the experiment. Thus the distribution of $k_{B}^{\text{field}}$ factors is influenced only by the ratios of the readings $\left(M_{Q2,B}^{\text{ref}}/M_{Q2,B}^{\text{field}}\right)\cdot \left(M_{Q1}^{\text{field}}/M_{Q1}^{\text{ref}}\right)$.

## 3 Materials and methods

### 3.1 Equipment

**Figure 1:**
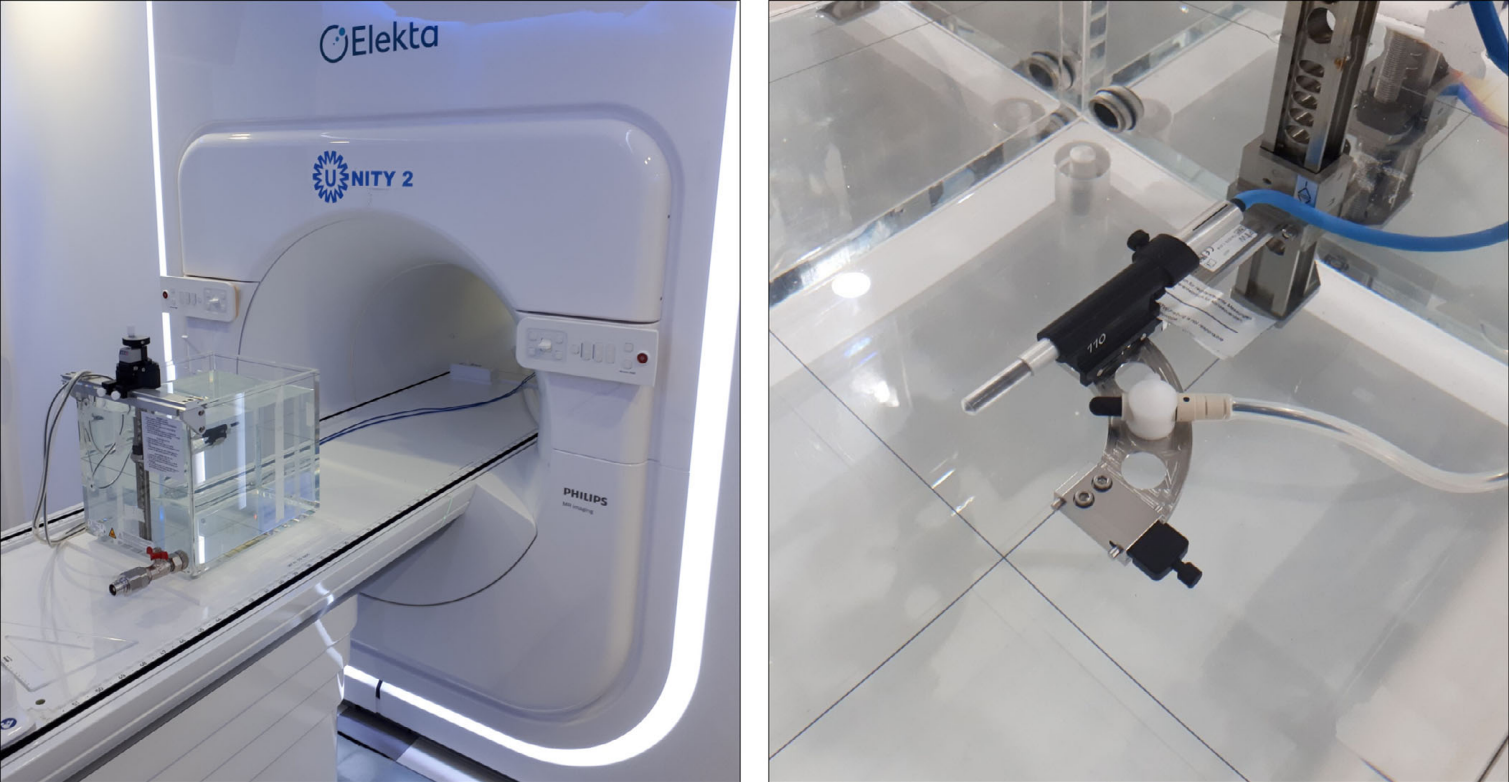
(a) PTW prototype MR‐compatible MP1 waterphantom at the magnetic resonance imaging (MRI)‐linac and (b) prototype holder, with PTW30013 chamber and CC13 monitor chamber. [Color figure can be viewed at http://wileyonlinelibrary.com]

For the most accurate and traceable results, measurements must be made in water.^4^, ^9^, ^11^ A PTW MP1 waterphantom with prototype MR‐compatible 1D manual drive and prototype Trufix BS right‐angled holder were used (PTW GmBH, Freiberg Germany). The setup is shown in Fig. 1.

Irradiation with *B* = 1.5 T was delivered with an Elekta Unity MRI‐linac using a 7 MV, flattening filter free, 10 × 10 cm^2^, 100 MU beam at ~430 MU/min with pulse repetition frequency (PRF) 275 Hz, gun duty cycle 71% and gantry 0^o^ (beam quality *Q*_2_). The position of the chamber was assessed, adjusted and verified using the rigid on‐gantry MV imager, such that the center of the chamber was at isocenter. The surface‐axis distance (SAD) of this system is 143.5 cm. The water level was then set to 10.0 cm above this point (SSD 133.5 cm). The linac was calibrated to deliver 0.720 cGy/MU under these conditions.

Irradiation with *B* = 0 T was delivered with a clinical Elekta Agility linac using a 6 MV, conventional flat, 10 × 10 cm^2^, 100 MU beam at ~500 MU/min with PRF 400 Hz, gun duty cycle 100% and gantry 0^o^ (beam quality *Q*_1_). The linac was calibrated to deliver 0.671 cGy/MU from this beam with SSD 100 cm and depth 10.0 cm, equivalent to ~0.81 cGy/MU under the conditions of this experiment (SSD 90.0 cm, depth 10.0 cm). For the Agility linac, the first chamber was setup with its center at isocenter using the room lasers, whose accuracy was previously confirmed through the standard departmental QA program. It is noted that, using another linac, it was not possible to exactly match the Unity beam quality and dose‐per‐pulse, but that the two inter‐comparisons were independent and therefore it was not critical that the conditions be perfectly matched.

Twelve PTW30013 ion chambers (serial numbers 5555, 5556, 5557, 5593, 5679, 5703, 5949, 5951, 5974, 5975, 8377 and 9627) were sourced from PTW and UMC Utrecht. Thirteen FC65‐G ion chambers (serial numbers 3642, 4077, 4081, 4082, 4083, 4090, 4091, 4092, 4093, 4095, 4096, 4097, 4098) were sourced from IBA and UMC Utrecht. In each case the construction of the set of chambers spanned more than 1 yr. The chambers were inspected visually and via 70 kVp x‐rays for any damage or faults in their electrodes or stems.

### 3.2 Measurements

Each time a new chamber was inserted the drive was left at the position corresponding to 10.0 cm above isocenter and the water level was checked against the center of the ion chamber by viewing the chamber and its reflection from just underneath the water surface, as per the method of TG‐106.^19^ This ensured that each chamber was placed as close to 10.0 cm depth as possible. In order to swap a chamber on the MRI‐linac, the table was withdrawn from the bore. On the Agility the table and phantom were never moved.

A single CC13 ion chamber (SN #5889, IBA‐Dosimetry, Schwarzenbruck Germany) was used as a monitor chamber throughout the experiment. The CC13 was mounted in a custom attachment on the same holder as the field chamber (see Fig. 1). The CC13 was connected to a PTW UnidosE T10009 electrometer with settings −250 V and medium range.

**Figure 2:**
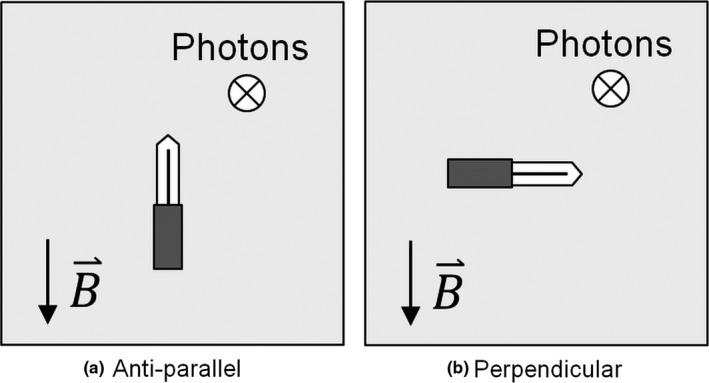
Gantry 0^o^ beam's eye views of the relative orientations of ion chamber, magnetic field, and beam direction for (a) antiparallel and (b) perpendicular (90 ^o^ counter clockwise) orientations.

Each field ion chamber was preirradiated with 400 MU and the collection voltage was applied for at least three minutes prior to nulling the chamber. Leakage was assessed over 30 s and initial measurements were monitored for drift. Measurements in the magnetic field were made with antiparallel (180^o^) and perpendicular (90^o^ counter‐clockwise) orientation (see Fig. 2), and with −250 V collecting potential. On the Agility linac, measurements were only made in one orientation (antiparallel) because the chamber sensitivity does not change with orientation since there is no magnetic field. Measurements were made with −250 V, −50 V and +250 V collecting potential in order to determine the recombination correction *k*_s_ and the polarity correction *k*_pol_ for each chamber. This was done to ensure that none of the chambers demonstrated unusual recombination or polarity behavior. At least five readings were made for each of the −250 V cross‐calibration conditions and at least three readings for the other voltages. Measurements were performed with a PTW UnidosE T10009 electrometer.

Recombination and polarity corrections do not depend on magnetic field and therefore the same values can be used for beams with and without magnetic field.^8^ Recombination does depend on dose per pulse which was 0.026 cGy/pulse for the MRI‐Linac and 0.017 cGy/pulse for the Agility. However, because the PTW30013 and FC65‐G are both standard Farmer chamber designs with very similar dimensions, the same relative change applies for both the local standard and field chambers and the ratios cancel out. This cancellation also applies for the volume averaging effect because, although the 7 MV FFF MRI‐linac and the 6 MV conventional Agility beams have different spatial dose distributions in the chamber volume, the differences apply equally to the local standard chamber and the field chamber.

Measurements were made with the local standard FC65‐G ion chamber SN 3129 at the beginning of each experiment. The −250 V measurements were repeated during the middle and at the end of each session.

### 3.3 Analysis

Each field chamber reading was normalized by the CC13 chamber reading in order to minimize the effect of any variations in the outputs of the linacs, as well as temperature and pressure variations.

The recombination correction was calculated using the two‐voltage method as recommended by NCS 18^9^ and IAEA TRS 398.^11^ The validity of this method in a 1.5 T MRI‐linac has been previously established via a Jaffe plot (1/*Q* vs 1/*V*).^8^ This was repeated for PTW30013 and FC65‐G chambers, which demonstrated linear behavior over (at least) the range 30–300 V (data not shown).

The polarity correction was calculated using the standard method.^9^, ^11^

The local standard chamber absorbed dose to water calibration coefficient, *N*_D,w,Q0_ = 4.827 cGy/nC, is traceable to (internationally accepted) primary measurement standards at the Dutch primary standards laboratory VSL (12 March 2018). The local standard chamber magnetic field correction factors, *k*_Bǁ_ = 1.0011 and *k*_B⊥_ = 0.9553, were determined previously by measurement on the Elekta Unity MRI‐linac with and without magnetic field (UMCU, 14 April 2018) using the method of van Asselen et al.^16^

Generic beam quality correction factors *k*_Q,Q0_ were determined for each chamber type from IAEA TRS 398^11^ using the measured Unity MRI‐linac and Agility linac *TPR*_20,10_ values of 0.709 and 0.681, respectively.

The multiple FC65‐G local standard measurements were used to assess the reproducibility of the experiment for a single chamber over the whole period. The field chamber results were calculated from the average local standard chamber measurements for each experiment.

For each PTW30013 and FC65‐G chamber, the *k*_B_ was calculated directly using Eq. (4). The inter‐chamber (intra chamber‐type) standard deviation of *k*_B_ was calculated, and evaluated against the calculated uncertainties of the experiment.

## 4 Results and discussion

No damage or faults in the electrodes or stem of each chamber were observed during visual and x‐ray inspection of the chambers.

Local standard chamber readings, corrected for the CC13 chamber, should be constant throughout the four experiment sessions. The standard deviations of the local standard chamber measurements were 0.11% and 0.10% (1.5 T), and 0.10% and 0.14% (0 T).

In this experiment there were several contributions to positional uncertainty. The reproducibility of chamber positioning depended on (a) the trufix BS holder (maximum measured difference 0.01 cm), (b) the MP1 manual drive accuracy (maximum measured difference 0.01 cm) and (c) the MRL table longitudinal accuracy (maximum measured difference 0.02 cm). Each time a new chamber was inserted any adjustment in water level was 0.02 cm or less.

Use of the CC13 monitor chamber on the same chamber holder as the field instruments meant that the CC13 chamber was subject to the same environmental conditions, with the potential exception of a small temperature gradient in the water. It also meant that, to first order, any residual error in holder depth would be cancelled out. Disadvantages included that (a) the chamber is smaller and therefore has lower signal‐to‐noise than a Farmer‐type chamber and (b) that it was placed approximately 3 cm off axis, which is in a dose gradient of 3%/cm in the FFF beam and could potentially transfer positional uncertainty into dosimetric uncertainty.

The statistical uncertainties (Type A) in the uncertainty budget for the distribution of magnetic field correction factors (Table 1) come from the standard deviations of the repeated local standard chamber measurements (*R*_Q_). Thus these include the positioning uncertainties. All the other entries are of type B, that is, based on calculations and estimates. Most are consistent with those of McEwen^18^ and de Prez.^13^ Uncertainties in variation in chamber angle and the difference in volume averaging are negligible. Uncertainty in $k_{B}^{\text{ref}}$ has no impact on the distribution of $k_{B}^{\text{field}}$ values, as $k_{B}^{\text{ref}}$ remains unchanged throughout, but it does affect the uncertainty of the final $k_{B}^{\text{field}}$ values. The recombination depends mostly on dose per pulse and varies little within a chamber type. Polarity varies little within chamber type. When applying generic *k*_Q_ factors from the codes of practice, the corrections are considered type‐specific rather than chamber specific. Here we consider ratios of these correction factors, limited within the same chamber‐type and for similar beam qualities (Q1 and Q2) with similar dose per pulse. Thus the uncertainties in ratios of recombination, polarity and beam quality for the same chamber type are expected to be small. In Table 1, estimates of these uncertainties are given on the basis of measured uncertainties in *k*_s_, *k*_pol_ and *k*_Q._ 13, 18

**Table 1:** Uncertainty budget (with coverage factor k = 1) in distribution of magnetic field correction factors represented by the uncertainty in $k_{B}^{\text{field}1}/k_{B}^{\text{field}2}$

Variable	Type	Agility B = 0 T *Q*_1_	Unity B = 1.5 T *Q*_2_
*R*_Q_ (nC/MU)	A	0.14%	0.11%
Recombination $\left(k_{s,Q1}^{\text{field}1}/k_{s,Q1}^{\text{field}2}\right)\times \left(k_{s,Q2}^{\text{field}2}/k_{s,Q2}^{\text{field}1}\right)$	B	<0.05%	<0.05%
Polarity $\left(k_{\text{pol},Q1}^{\text{field}1}/k_{\text{pol},Q1}^{\text{field}2}\right)\times \left(k_{\text{pol},Q2}^{\text{field}2}/k_{\text{pol},Q2}^{\text{field}1}\right)$	B	<0.05%	<0.05%
Ratios of *k*_PT_ and humidity correction	B	0.03%	0.03%
Ratio $k_{Q2,Q1}^{\text{field}2}\times k_{Q1,Q2}^{\text{field}1}$	B	<0.12%	
Combined standard uncertainty (k = 1) in $k_{B}^{\text{field}1}/k_{B}^{\text{field}2}$		0.24%	

**Figure 3:**
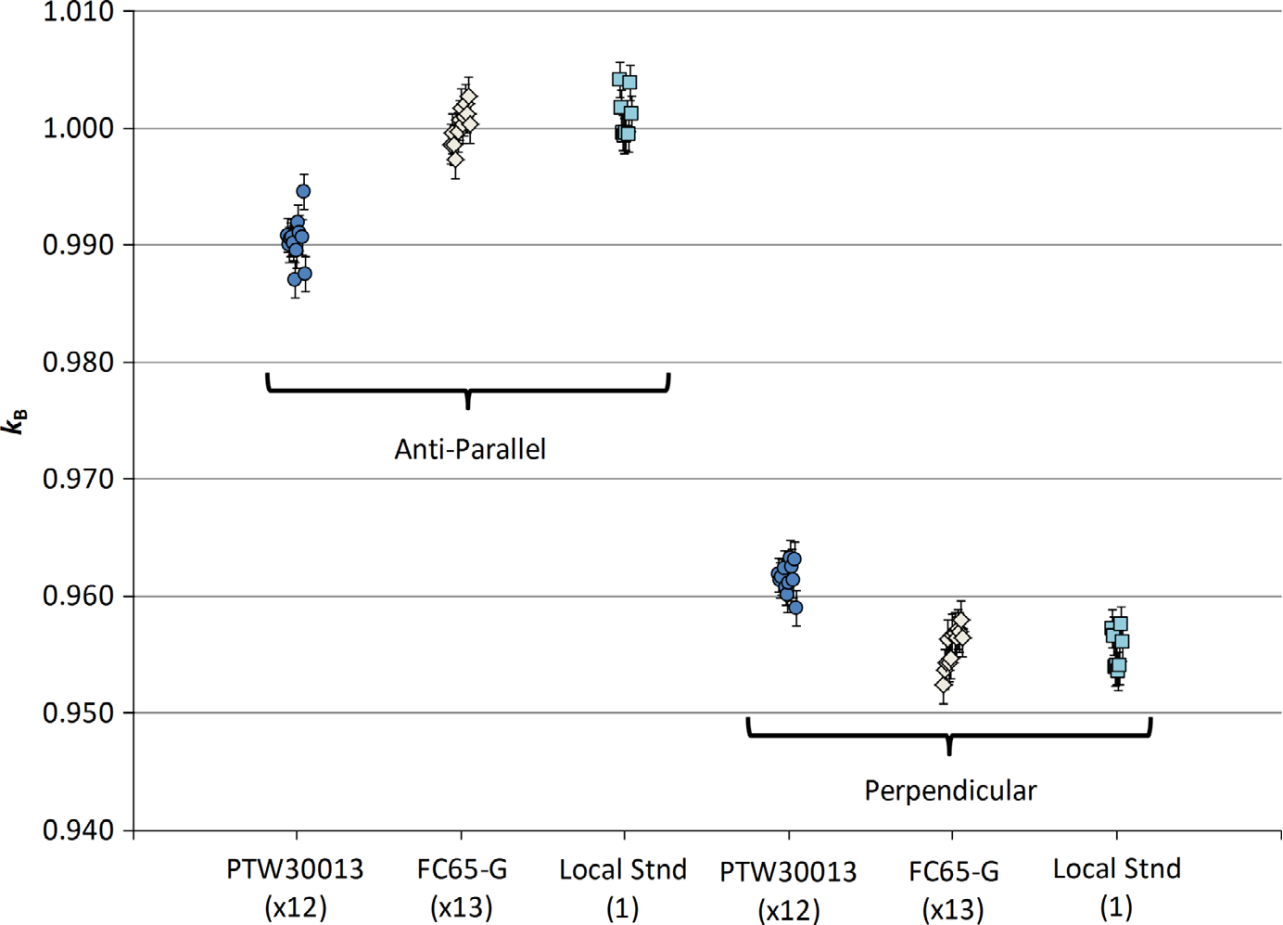
Magnetic field correction factors (*k*_B_) for all the PTW30013 (circles) and FC65‐G (diamonds) chambers in both parallel and perpendicular orientations. Within each chamber type and orientation, the results are very consistent. For comparison, the results of repeated measurements of a single local standard chamber are shown (squares). The variation in each set of chambers is no greater than the variation in measurements of a single chamber. [Color figure can be viewed at http://wileyonlinelibrary.com]

The *k*_B_ results for the twelve PTW30013 chambers and the thirteen FC65‐G chambers are shown in Fig. 3 and summarized in Table 2.

**Table 2:** Averages and standard deviations of *k*_B_ for PTW30013 and FC65‐G chamber types

	*k*_Bǁ_		*k*_B⊥_	
	PTW30013	FC65‐G	PTW30013	FC65‐G
Average	0.990	1.000	0.961	0.956
Standard deviation (%)	0.19%	0.15%	0.13%	0.17%

The goal of this study was to assess the standard deviations of the magnetic field correction factors for the two chamber types to determine if type‐specific generic *k*_B_ values are valid. Thus the key results in Table 2 are the standard deviations for *k*_B_: 0.19% (ǁ) and 0.13% (⊥) for the PTW30013 and 0.15% (ǁ) and 0.17% (⊥) for the FC65‐G. These values are all less than the standard uncertainty of 0.24% calculated in the uncertainty budget. Thus the observed distributions of magnetic field correction factors for the PTW30013 and FC65‐G chambers are equivalent to the variation expected due to measurement uncertainty. This implies that it is valid to use a generic chamber‐type magnetic field correction factor for either of these chamber types used in a specific field‐beam‐chamber orientation.

The *k*_B_ factors for each of the PTW30013 and FC65‐G chambers were determined from an existing *k*_B_ for the local standard chamber. The average results for PTW30013 were *k*_Bǁ_ = 0.990 and *k*_B⊥_ = 0.961 and for FC65‐G *k*_Bǁ_ = 1.000 and *k*_B⊥_ = 0.956, all ± 0.004 (k = 1). The uncertainty in these values is greater because they include the uncertainty of 0.28% (k = 1) in the local standard chamber factor $k_{B}^{\text{ref}}$.

It may also be useful to compare between the magnetic field correction factors in the two directions. The ratios of k_Bǁ_/k_B⊥_ are 1.030 ± 0.001 for PTW 30013 and 1.047 ± 0.001 for FC65‐G. The k_Bǁ_ and k_B⊥_ measurements are correlated for each chamber type (Pearson correlation coefficient r = 0.88 for PTW30013 and 0.65 for FC65‐G). The parallel and perpendicular measurements were acquired consecutively without making any adjustments to depth or water level, therefore it is not a surprise that the observed standard uncertainties (k = 1) are smaller for the ratios (k_Bǁ_/k_B⊥_). This result implies that if the *k*_B_ values for an individual chamber are desired, it is sufficient to check the individual chamber in only one orientation.

The *k*_B_ results in this study are based on measurements that are entirely separate from, but consistent with, previously published results shown in Table 3.3 The van Asselen et al. results were derived from a smaller number of chambers, but with multiple magnetic field ramp ups and ramp downs. Thus they are likely to have a smaller systematic error in *k*_B_ than the results presented here. van Asselen et al. also contains comparisons to results from Monte Carlo based studies which are broadly consistent.

**Table 3:** Comparison of results from this study (combined standard uncertainties k = 1) with those from van Asselen et al.^16^ (type A standard uncertainties). Results are consistent

Chamber type	Reference	*k*_Bǁ_	*k*_B⊥_
PTW30013	This study	0.990 ± 0.004	0.961 ± 0.004
	van Asselen	0.992 ± 0.002	0.963 ± 0.002
FC65‐G	This study	1.000 ± 0.004	0.956 ± 0.004
	van Asselen	0.997 ± 0.003	0.952 ± 0.002

The variation in the measured magnetic field correction factors is approximately the same for both anti‐parallel and perpendicular orientations. Nevertheless, the anti‐parallel factors are relatively close to unity (i.e., minimal perturbation) and therefore the parallel orientation is preferable to the perpendicular orientation. In addition the parallel and anti‐parallel orientations can be more easily used for beams from different gantry angles and thus this setup is more practical for other dosimetry purposes (e.g., treatment plan QA).

The recombination and polarity corrections were almost identical within both chamber types. For the PTW30013 chambers *k*_s_ = 1.003 (σ 0.01%) and *k*_pol_ = 0.999 (σ 0.02%). For the FC65‐G chambers *k*_s_ = 1.004 (σ 0.02%) and *k*_pol_ = 1.000 (σ 0.03%). The derived *N*_D,w,Q0_ factors had standard deviations of 0.31% (PTW30013) and 0.32% (FC65‐G). As a consistency check the values derived here for the three UMCU chambers were compared to their previous values. The new *N*_D,w,Q0_ factors were different by −0.03%, +0.05% and −0.20%.

It has been noted in the literature^13^, ^18^ that differences of up to 0.4% exist at 6–7 MV in estimates of generic (chamber‐type) *k*_Q,Q0_ factors between direct measurements (e.g., NCS 18^9^) and generic‐type calculations (e.g., IAEA TRS 398^11^). This can affect calculations of cross‐calibration parameters such as *N*_D,w,Q0_. However the method of deriving magnetic field correction factors used here is insensitive to *k*_Q,Q0_ values as the ratios largely cancel.

The effective point of measurement (EPoM) of the Farmer‐type ion chamber is shifted laterally and vertically due to the spiraling trajectories of the electrons. The combination of electric and magnetic fields also creates nonuniform sensitivity within the nominal chamber volume.^14^, ^20^ The kB factor includes the effect of the change in EPoM due to the magnetic field.

## 5 Conclusion

The goal of this study was to assess the consistency of magnetic field correction factors within the PTW30013‐type and FC65‐G‐type ion chambers. The factors were found to be very consistent, with observed standard deviations of σ = 0.13%–0.19%, which were within the standard measurement uncertainty of 0.24% (k = 1) from the uncertainty budget. These results indicate that it is not necessary to derive a new factor for every new PTW30013 or FC65‐G chamber, but that average values for each chamber‐type (with careful attention to beam energy, magnetic field strength and beam‐field‐chamber orientations) can be applied from the literature.

## Conflict of interest

The authors have no conflicts to disclose.
